# The role of job strain in the relationship between depression and long-term sickness absence: a register-based cohort study

**DOI:** 10.1007/s00127-024-02700-7

**Published:** 2024-06-25

**Authors:** Rand Jarroch, Daniel Falkstedt, Alicia Nevriana, Kuan-Yu Pan, Jussi Kauhanen, Melody Almroth

**Affiliations:** 1 Institute of Public Health and Clinical Nutrition, University of Eastern Finland, Kuopio, Finland; 2 Institute of Environmental Medicine, Karolinska Institute, Stockholm, Sweden; 3 Department of Public Health Sciences, Stockholm University, Stockholm, Sweden

**Keywords:** Depression, Long-term sickness absence, Job strain, Job control, Job demands, Swedish workers

## Abstract

**Purpose:**

Though individuals with depression and those with poor working conditions are more likely to be on long-term sickness absence (LTSA), less is known about how working conditions may modify the associations between depression status and LTSA. This study aims to examine the association between depression and LTSA among Swedish workers with different levels of job strain and its individual components (job demands and job control).

**Methods:**

All Swedish workers 30 − 60 years old (*N* = 3,065,258) were studied in 2005. At baseline (2005–2010), workers were categorized as: without depression, being prescribed antidepressants, and being in inpatient/outpatient care. Job strain was measured using a Swedish Job Exposure Matrix, and data on LTSA were obtained from 2011 to 2021. The association between depression and LTSA was assessed using Cox proportional-hazards regression stratified by categories of job strain.

**Results:**

Compared to workers without depression, workers with depression had higher risk of LTSA across all job strain levels. Depression was associated with the highest hazards of LTSA in active jobs, but a similar population attributable fraction (PAF) was found across categories of job strain, indicating similarities between the different categories.

**Conclusion:**

There was evidence of a moderating effect of job strain in the relationship between depression and LTSA, but also evidence that this was due to differences in baseline depression prevalence in the different job strain categories. Future research is needed to determine alternative factors which could be relevant for reducing LTSA among those who have already developed depression.

**Supplementary Information:**

The online version contains supplementary material available at 10.1007/s00127-024-02700-7.

## Introduction

Long-term sickness absence (LTSA) poses a serious public health challenge and socioeconomic burden to many countries in Europe [1, 2]. Mental health problems such as depression are important risk factors for LTSA and delay in return to work (RTW) which could lead to permanent exclusion from the labor market [3–5]. Working conditions have been suggested as an important factor which could influence the occurrence and duration of LTSA [6]. Therefore, modifiable factors at the workplace may be a target to intervene to lower the risk of LTSA among people with depression.

Adverse psychosocial working conditions have been found to predict sickness absence (SA) [7] and its underlying triggers such as mental health problems [8]. A common way of measuring factors related to the psychosocial work environment is through the job strain model first introduced by Karasek [9], which categorizes the work environment based on combinations of job control and demands. The job strain model divides work conditions into four categories: high strain jobs (high demands/ low control), low strain jobs (low demands/ high control), active jobs (high demands/ high control) and passive jobs (low demands/ low control). High strain jobs and passive jobs have both been found to be associated with adverse health outcomes [10–12] and risk of SA [13]. Active jobs have also been found to be associated with an increased risk of SA [7], but also to be protective of health along with low strain jobs [14, 15].

Although there is a vast body of evidence showing that adverse working conditions are related to worse mental health and higher rates of SA [16], less is known about how job strain may modify the likelihood of developing LTSA among those with previous mental health problems such as depression. This may be especially important given that there is evidence that later RTW from those on SA for common mental disorders is associated with worker’s dissatisfaction with their working conditions [3, 17].

In Sweden, the total costs of SA for the Swedish social security system were close to €11.28 billion in 2010 corresponding to 4% of the gross domestic product (GDP) and they include sickness benefits and costs of rehabilitation [18]. Workers in active jobs had a high increase in sickness days compared to other job strain levels [19]. Another study found that high strain jobs increased the odds for LTSA among Swedish women and men, while active jobs had an increased risk of LTSA among women [7]. It has been estimated in OECD countries including Sweden that among the working age population, approximately 5% suffer from severe mental disorders, and another 15% have mild to moderate mental disorders [20], of which depression is one of the most common conditions. Yet, very little is known about how job strain might affect LTSA among workers who already have depression. To our knowledge, there are no previous studies that investigated the effect modification of job strain on LTSA among workers with depression.

This register-based cohort study aims to examine the association between previous depression status and LTSA among Swedish workers with different levels of job strain and its components (job demands and job control). Our main hypothesis is that associations between depression status and the prospective risk of LTSA will be weaker among the more favorable working conditions such as in low strain and active jobs.

## Methods

### Study population

This study used data from the Swedish Work, Illness, and labor-market Participation (SWIP) cohort [12, 21]. The SWIP cohort includes all individuals (around 5.4 million people) who were 16–64 years old and registered as living in Sweden in 2005. The cohort was created through linkages of several registers using the Swedish personal identity numbers and the data were obtained and de-identified by Statistics Sweden. For this study, data from the Swedish total population register, the Longitudinal Integrated Database for Health Insurance and Labour Market Studies (LISA) register, the occupations register, the Micro Data for Analysis of the Social Insurance System (MIDAS) register, the Swedish national patient register and the prescribed drug register were used. Workers born between 1949 and 1979 (30–60 years old in 2009) were selected for this study (*N* = 3,725,143). This restriction was made because it is assumed that those in this age group would have been both established and remaining in the labor market. We excluded those with previous disability pension (*N* = 384,138), those who were missing an occupational title during all years from 2005 to 2009 (*N* = 261,130), and those with missing data on any other covariate (*N* = 14,617). After exclusions, a total of 3,065,258 workers (51.72% men and 48.28% women) were included in the study.

Ethical approval was obtained from the Stockholm ethics review board, reference number 2017/1224-31 and 2018/1675-32.

### Exposure

Workers in this study were divided into three groups based on depression status between the years 2005 and 2009 as follows: workers without registered treatment for depression; workers who were prescribed antidepressants only; and workers with inpatient or outpatient care for depression. The inpatient register provides information on hospitalizations while the outpatient register provides information on specialized care for depression using the international classification of disease (ICD) codes F32 and F33. Data on the use of antidepressants were obtained from the prescribed drug register using the anatomical therapeutic chemical classification system (ATC) code category NO6A.

### Outcome

Data on LTSA were obtained from the MIDAS register. LTSA was defined as a sickness absence spell of at least 90 days during the follow up period (2010–2021) due to any diagnosis. Data on LTSA due to psychiatric diagnosis (ICD F chapter) were further obtained.

### Effect modifiers

Job control and job demands were measured using a Job Exposure Matrix (JEM) based on the Swedish Work Environment and Health surveys where a random sample of around 10,000 individuals every 2 years are contacted [22]. Around 90,000 people responded for the period of 1997 to 2013. These surveys measure the aggregated experience of different exposures within 355 occupations based on the Swedish version of the ISCO-88 (International Standard Classification of Occupations), separately for men and women. These average exposures are then linked to the index individuals based on the same occupational code registered in each year from 2005 to 2009. Job control was measured using four items assessing decision authority, which indicates the amount of influence that an individual has in the way their work is done. Job demands were measured using three items focused on the stress, time and level of concentration of the job. An average over the five years 2005 − 2009 was calculated and variables were dichotomized using the median as the cutoff point for men and women separately [12]. Job strain was measured by combining job control and job demands split at their medians to make four quadrants (high control/low demands (low strain), high control/high demands (active), low control/low demands (passive) and low control/high demands (high strain) [9]. A previous version of this JEM containing the same questions concluded that the JEM was valid for use in the Swedish population [22]. When comparing the JEM data with the individual responses in this validation study, correlation coefficients were around 0.40 for job control for men and 0.44 for women and 0.26 for demands for men and 0.30 for women. These estimates are also in line with a Finnish study using a JEM measuring similar exposures which concluded that JEMs measuring job strain had high validity and could be used reliably when individual-level data is not available [23]. Because JEMs are a method for avoiding common methods and self-report bias, JEM and individual response data are not expected to be identical. Validation using outcome data (external validity) has therefore been used in previous studies and has been judged to be satisfactory [22, 23].

### Covariates

Several variables were taken from the LISA register from 2005 [24]. Educational attainment was divided into three groups: (i) primary (≤ 9 years); (ii) secondary (10–12 years); and (iii) tertiary (≥ 13 years) which corresponds to university-level education. Civil status was categorized as either married, unmarried, divorced, or widowed. Country of birth was dichotomized to indicate whether an individual was born inside or outside of Sweden. Unemployment days five years before the start of follow-up (2000–2005) were divided into three groups: (i) no unemployment (ii) 1–365 days of unemployment and (iii) > 365 days of unemployment. From the MIDAS register, we obtained data on MSD sick leave of at least 14 days prior to 2010 and any LTSA of at least 90 days prior to 2010. Physical workload was estimated using a JEM based on eight questions that involve heavy lifting, uncomfortable working postures, repetitive work and physically demanding work, and was dichotomized at the median.

### Statistical analysis

To characterize the workers with different levels of depression, we calculated the distribution of covariates across the three categories of depression status (no depression, prescribed antidepressants and inpatient/outpatient care). We also estimated the crude associations between covariates and all-cause LTSA using Cox proportional-hazards regression with age as the underlying time scale.

Cox regression models were built to estimate associations between depression status and LTSA stratified by job strain categories (high strain, low strain, active jobs, passive jobs), as well as job demands and job control separately. The proportional hazards assumption was examined visually using Kaplan-Meier curves showing risk of LTSA in each depression group over time. No violation was detected. Model 1 shows the crude associations (with age as the underlying time scale) and Model 2 is adjusted for birth year, gender, education, civil status, country of birth, unemployment, previous MSD-SA and physical workload. Person-time was calculated from 1 January 2010 until either emigration, disability pension, turning 65 years old, death, LTSA or the end of follow-up (Dec 31, 2020). In addition to the stratified models, effect modification was tested by including interaction terms between depression and job strain (or its individual components). We used a likelihood ratio test to test the overall significance of the interaction where a *p*-value of < 0.05 corresponded to a modified association between depression and LTSA. To account for the difference in prevalence of depression across strata, we calculated the population attributable fraction (PAF) for each category of depression status across each stratum of job strain and its separate components. This was done using Miettinen’s formula (PAF = Pc X (HR-1/HR) where Pc = proportion of LTSA cases exposed to depression status) [25].

Because LTSA due to psychiatric diagnoses is arguably the most relevant diagnoses in relation to prior depression diagnoses, and to investigate whether results may have been driven by other less relevant diagnoses, we repeated the regression analyses while only considering LTSA due to a psychiatric diagnosis. We also repeated the regression analyses for women and men separately to investigate any potentially important gender differences.

Finally, to further investigate whether adjusting for previous MSD-SA provided adequate control for confounding, we performed the same analyses after excluding those with an SA period of more than 90 days due to any cause prior to 2010.

All analyses were conducted using SAS Enterprise Guide 8.3 Fourth (SAS Institute, Cary, NC, USA).

## Results

### Characteristics of the study population

The distribution of covariates according to depression status at baseline is shown in Table 1. Around 12.3% of the workers were prescribed antidepressants or diagnosed with depression in in- or outpatient care during the baseline years. Those with inpatient/outpatient care tended to be slightly younger compared to the other groups. More women received inpatient/outpatient care, and especially antidepressants. Those treated for depression were more often born outside of Sweden, lower educated, and less likely to be married, especially among those treated in inpatient/outpatient care. Those who were treated for depression were also more likely to be in passive jobs and less likely to be in active jobs and were more often in low demand, low control, and high physical workload jobs, especially among those who received inpatient/outpatient care. Finally, those treated for depression had more unemployment days and previous sick leave due to MSD, the latter of which was most common among those treated with antidepressants.

**Table 1:** Distribution of baseline covariates according to baseline depression status

	No depression treatment	Antidepressant treatment	In/outpatient treatment
Total	2,689,266 (87.73)	323,994 (10.57)	51,998 (1.7)
Age Mean (SD)	44.53 (8.71)	45.12 (8.51)	43.04 (8.48)
Gender N (%)			
Men	1,445,327 (53.74)	118,327 (36.55)	21,661 (41.66)
Women	1,243,939 (46.26)	205,622 (63.46)	30,337 (58.34)
Country of birth N (%)			
Sweden	2,344,419 (87.18)	276,278 (85.41)	42,936 (82.57)
Other	344,847 (12.82)	47,266 (14.59)	9062 (17.43)
Educational level N (%)			
Primary (≤ 9 years)	300,024 (11.16)	40,061 (12.36)	6977 (13.42)
Secondary (10 − 12 years)	1,311,359 (48.76)	163,120 (50.35)	25,998 (50)
Tertiary (≥ 13 years)	1,077,883 (40.08)	120,813 (37.29)	19,023 (36.58)
Civil status N (%)			
Married	1,234,402 (45.90)	139,610 (43.09)	18,177 (34.96)
Unmarried	1,173,948 (43.65)	133,131 (41.09)	25,042 (48.16)
Separated	267,798 (9.96)	48,806 (15.06)	8461 (16.27)
Widowed	13,118 (0.49)	2447 (0.76)	318 (0.61)
Job strain N (%)			
Active job	847,014 (31.50)	84,685 (26.13)	11,253 (21.64)
High strain	518,404 (19.28)	63,603 (19.63)	10,388 (19.98)
Low strain	515,173 (19.16)	65,255 (20.14)	10,182 (19.58)
Passive job	808,675 (30.07)	110,451 (34.09)	20,175 (38.80)
Job demands N (%)			
High	1,365,418 (50.77)	148,288 (45.77)	21,641 (41.62)
Low	1,323,848 (49.23)	175,706 (54.23)	30,357 (58.38)
Job control N (%)			
Low	1,327,079 (49.35)	175,054 (53.72)	30,563 (58.78)
High	1,362,187 (50.65)	149,940 (46.28)	21,435 (41.22)
Physical workload N (%)			
Low	1,373,939 (51.09)	150,745 (46.53)	21,341 (41.04)
High	1,315,327 (48.91)	173,249 (53.47)	30,657 (58.96)
Unemployment (days) N (%)			
0	2,119,452 (78.81)	223,594 (69.01)	29,122 (56.01)
1–365	435,264 (16.19)	72,964 (22.52)	16,215 (31.18)
> 365	134,550 (5.00)	27,436 (8.47)	6661 (12.81)
Previous sick leave due to MSD			
No	2,425,696 (90.20)	262,324 (80.97)	43,363 (83.39)
Yes	263,570 (9.80)	61,670 (19.03)	8635 (16.61)

Regardless of depression treatment history, women, those born outside of Sweden, those with lower education and those who were divorced or widowed had an increased risk of LTSA (Table 2). In addition, workers in high strain, passive, low control, and high physical workload jobs had an increased risk of LTSA while those in active jobs and low demands jobs had a decreased risk of LTSA. Previous unemployment and previous MSD or long-term all-cause SA were associated with higher risks of LTSA as well.

**Table 2:** The univariate association of covariates and long-term sickness absence

Variable	Category	Hazards ratio (95% confidence interval)
Gender	Men	1.00
	Women	1.75 (1.75 − 1.77)
Country of birth	Sweden	1.00
	Other	1.15 (1.14 − 1.16)
Educational level	Tertiary (≥ 13 years)	1.00
	Secondary (10 − 12 years)	1.34 (1.33 − 1.35)
	Primary (≤ 9 years)	1.54 (1.53 − 1.55)
Civil status	Married	1.00
	Unmarried	0.99 (0.99 − 1.00)
	Divorced	1.36 (1.35 − 1.38)
	Widowed	1.28 (1.24 − 1.32)
Job strain	Low strain	1.00
	Active job	0.69 (0.68 − 0.69)
	High strain	1.03 (1.02 − 1.03)
	Passive job	1.19 (1.18 − 1.20)
Job demands	Low	1.00
	High	0.73 (0.72 − 0.73)
Job control	High	1.00
	Low	1.40 (1.39 − 1.41)
Physical workload	Low	1.00
	High	1.59 (1.58 − 1.59)
Unemployment (days)	0	1.00
	1–365	1.45 (1.44 − 1.45)
	> 365	1.52 (1.51 − 1.54)
Previous sick leave due to MSD	No	1.00
	Yes	2.56 (2.54 − 2.57)
Previous long term sick leave	No	1.00
	Yes	2.98 (2.96 − 2.99)

### Association between depression and LTSA according to job strain, job demands and job control

Workers with previous treatment of depression had an increased risk of LTSA regardless of working conditions, especially for those treated in inpatient/outpatient care (Table 3). Associations between depression status and LTSA tended to be strongest among those in active jobs (HR 2.80 95% CI 2.76–2.84 for the antidepressant group and HR 4.41 95% CI 4.29–4.54 for inpatient/outpatient group) and weakest among those in passive jobs (HR 2.30 95% CI 2.28–2.32 for the antidepressant group and HR 3.12 95% CI 3.05–3.18 for the inpatient/outpatient group). Similarly, associations were stronger in high demand and high control jobs compared to low demand and low control jobs. Estimates were attenuated after adjusting for covariates, but the pattern remained the same. All interaction terms between depression and job strain, job control, and job demands were statistically significant.

**Table 3:** Associations between depression status and LTSA stratified by job type

		Cases of LTSA				
Job strain		*N* (%)	Model 1	Model 2	LR test	PAF
Active job	No depression	118,355 (13.97)	1.00	1.00		
	Antidepressants	28,394 (33.53)	2.80 (2.76 − 2.84)	2.31 (2.28 − 2.34)		10.97%
	In/outpatient care	5085 (45.19)	4.41 (4.29 − 4.54)	3.60 (3.50 − 3.70)		2.97%
High strain	No depression	105,601 (20.37)	1.00	1.00		
	Antidepressants	25,458 (40.06)	2.33 (2.30 − 2.36)	2.05 (2.02 − 2.08)		9.94%
	In/outpatient care	5208 (50.13)	3.43 (3.33 − 3.52)	3.06 (2.98 − 3.15)		3.16%
Low strain	No depression	102,056 (19.81)	1.00	1.00		
	Antidepressants	25,933 (39.74)	2.39 (2.35 − 2.42)	1.99 (1.97 − 2.02)		10.07%
	In/outpatient care	5094 (50.03)	3.59 (3.49 − 3.69)	3.02 (2.93 − 3.11)		3.17%
Passive job	No depression	187,036 (23.13)	1.00	1.00		
	Antidepressants	48,245 (43.68)	2.30 (2.28 − 2.32)	1.93 (1.90 − 1.94)		9.82%
	In/outpatient care	10,150 (50.31)	3.12 (3.05 − 3.18)	2.74 (2.68 − 2.79)	< 0.0001	3.26%
Demands						
High	No depression	223,956 (16.40)	1.00	1.00		
	Antidepressants	53,852 (36.32)	2.61 (2.58 − 2.63)	2.19 (2.17 − 2.21)		9.44%
	In/outpatient care	10,293 (47.56)	4.01 (3.93 − 4.09)	3.34 (3.28 − 3.41)		2.84%
Low	No depression	289,093 (21.84)	1.00	1.00		
	Antidepressants	74,178 (42.22)	2.33 (2.32 − 2.35)	1.95 (1.93 − 1.97)		11.09%
	In/outpatient care	15,244 (50.22)	3.28 (3.23 − 3.34)	2.83 (2.79 − 2.88)	< 0.0001	3.51%
Control						
Low	No depression	292,636 (22.05)	1.00	1.00		
	Antidepressants	73,703 (42.34)	2.32 (2.30 − 2.34)	1.97 (1.95 − 1.98)		9.85%
	In/outpatient care	15,358 (50.25)	3.23 (3.18 − 3.29)	2.84 (2.79 − 2.89)		3.23%
High	No depression	220,413 (16.18)	1.00	1.00		
	Antidepressants	54,327 (36.23)	2.64 (2.61 − 2.66)	2.15 (2.13 − 2.17)		10.57%
	In/outpatient care	10,179 (47.49)	4.09 (4.01 − 4.18)	3.30 (3.23 − 3.36)	< 0.0001	3.07%

LTSA: Long term sickness absence

Model 1: Adjusted for age

Model 2: Adjusted for age, gender, education, civil status, country of birth, unemployment prior to the start of follow-up, diagnosis of musculoskeletal disorders prior to the start of follow-up, physical workload prior to the start of follow-up

LR test: Likelihood ratio test of interaction

PAF: Population Attributable Fraction = Pc X (HR-1/HR) where Pc = proportion of LTSA cases exposed to depression treatment

The PAF was similar for antidepressants and inpatient/outpatient care respectively across strata of job strain, job demands and job control (PAF ranging from 9.82 to 10.97% for antidepressants and 2.97–3.26% for inpatient/outpatient care across strata of job strain).

### Additional analyses

In adjusted models with the outcome of psychiatric LTSA rather than all cause LTSA, estimates for the association between antidepressant prescription and psychiatric LTSA were similar across categories of job strain, job demands, and job control (Table S1). Associations between inpatient/outpatient depression treatment and psychiatric LTSA were slightly stronger in the low strain and passive job groups, but confidence intervals were overlapping.

Though LTSA was generally more common among women, associations between depression status and LTSA tended to be stronger among men compared to women (Table S2 and S3). The strongest associations between depression status and LTSA were found in the categories of active jobs, and high demand and high control jobs for both men and women.

When excluding those with LTSA prior to baseline, associations were weaker than the main results, but showed the same patterns, indicating that observed associations were not completely explained by previous LTSA (Table S4).

## Discussion

### Findings of the study

This study investigated the possible modifying impact of job strain, job demands and job control on the risk of LTSA (at least 90 days) among Swedish workers treated for depression compared to Swedish workers without depression. We found that workers treated for depression had higher risks of all-cause LTSA compared to workers without depression across all job categories. We found evidence for significant interaction in the relationship between depression and job strain and LTSA. Contrary to our hypothesis, however, we found that associations between depression status and LTSA were strongest among those in active jobs. On the other hand, the proportion of LTSA that could be attributed to depression status was similar across job categories, and associations were similar across strata when only considering LTSA due to psychiatric diagnoses. Conclusions were similar when considering men and women separately, and when excluding those with previous LTSA.

### Comparison with the previous literature

It is well-established that depression is associated with elevated risk of LTSA, which increases among the more severe cases [26]. This was clearly observed in our study as workers with depression had higher risk of LTSA compared to workers without depression, and the risk increased proportionally with the severity of depression (workers with inpatient/outpatient care had even higher risk of LTSA than workers prescribed antidepressants).

Previous studies have found that the quadrants of the demand-control model with low job control were consistent predictors of SA [27–29]. However, it has also been found that low job control and high job demands measured separately are associated with higher all-cause and musculoskeletal SA but not to SA due to psychiatric diagnoses [30].

In our analysis of the baseline characteristics of our study population, we observed that those in passive jobs, and thus, in low demand and low control jobs were more likely to be treated for depression at baseline. Additionally, passive jobs in general were associated with an increased risk of LTSA during follow-up. Both of these findings were in line with previous studies which showed increased risks for depression [12] and higher SA [13] among those in passive jobs. However, in the present study, associations between depression status and LTSA tended to be weakest in passive jobs and strongest in active jobs, which was contrary to our hypothesis. Since we are not aware of previous studies which investigated the possible effect modification by working conditions of the relationship between depression and LTSA, these findings are somewhat novel and difficult to compare with previous studies.

### Interpretations of the results

Taken on its own, the finding that associations between depression status and LTSA were stronger among those in active jobs, as well as high control and high demands jobs separately, it could be interpreted that active jobs lead to a greater risk of LTSA among those treated for depression. This could either be due to the work environment itself or to greater access to social benefits such as sickness absence among those in active jobs. In fact, a recent publication using a similar population found that those with lower quality employment were less likely to use sickness absence benefits, attributing this finding to presenteeism [31]. However, given that LTSA was generally lower among those in active jobs across levels of depression status, these explanations seem less likely.

Those in active jobs were less likely to have been treated for depression at baseline, while those in passive jobs were most likely to have been treated for depression. Similarly, those in active jobs had the lowest overall risk for LTSA during follow-up, while those in passive jobs had the highest risk. The comparison between those treated for depression and those not treated for depression therefore showed the weakest contrast in LTSA among those in passive jobs and the greatest contrast among those in active jobs due to a higher overall incidence of LTSA in the passive job group regardless of depression status. That the PAF was similar across the different job categories for both antidepressant and inpatient/outpatient treatment also indicates that when taking the initial prevalence of depression status in each job group into account, there is little difference between the groups in terms of how much LTSA can be attributed to previous occurrence of depression.

When only considering LTSA with a psychiatric diagnosis, estimates across job types were more similar to each other than when considering all-cause LTSA. In other words, when removing LTSA cases that were not directly related to mental disorders, the associations between depression status and LTSA due to a psychiatric diagnosis were similar across the different job types. This further supports that other LTSA diagnoses, such as musculoskeletal diagnoses, are more common in passive jobs regardless of depression status. If more individuals in the reference group (those without depression) are more likely to have musculoskeletal diagnoses and therefore a higher risk of LTSA, then relative risks will be smaller.

For the gender-specific analysis, associations tended to be stronger for men, which indicates that there is a greater contrast of LTSA for men between those with and without depression. LTSA and depression were more common among all groups of women. There are therefore probably more unmeasured risk factors for LTSA among women, leading to less contrast in comparison groups.

### Strengths and limitations

The main strength of our study is the large study population that included around three million Swedish workers. This in turn allowed us to comprehensively investigate the role of several variables such as gender, previous LTSA and associated diagnosis in the associations found between depression at baseline and LTSA during follow-up. It further allowed the adjustment for differences in age, education level, marital status, MSD diagnosis, physical workload and history of unemployment before baseline which we consider as reasonable control for confounding. Furthermore, studying the entire population limits issues of selection and attrition bias. Another strength is the use of a JEM for ranking individuals within the study population in terms of job strain. The use of JEMs reduces differential misclassification that might occur when using self-reported data on psychosocial factors at work [22].

Using JEMs, however, also has limitations as it reflects mean values which measure differences between occupations but not between individuals in the same occupation. This can result in non-differential misclassification and downward bias of risk estimates. On the other hand, relying on the same informant to assess both work environment and mental health has been described as leading to biased and inflated associations compared to using JEMs [32].

Another limitation of the study is that we did not have information on depression diagnoses from primary care. Using antidepressant prescriptions and inpatient and specialized care was a strategy to try to capture as accurate information on depression as possible. However, since inpatient/outpatient care is limited to more severe cases and antidepressants are often prescribed for diagnoses other than depression such as anxiety, there is a risk for misclassification of the exposure which we assume to be non-differential. Finally, depression status and occupational exposures were measured during the same five-year period of time, which makes it difficult to interpret the temporal order of events. This difficulty is likely to apply to other studies of chronic disorders and working conditions in particular.

## Conclusions

This study found depression to be strongly related to long-term sickness absence (LTSA) during follow-up regardless of levels of job strain and its specific components (job demand and job control). Associations appeared stronger among those in active jobs but considering the lower prevalence of individuals with depression in this group and the lower incidence of LTSA, we conclude that these differences should be interpreted with caution. Future research is needed to determine alternative factors which could be relevant for reducing LTSA among those who have already developed depression.

## Electronic Supplementary Material

Below is the link to the electronic supplementary material.

Supplementary Material 1
