# Supplementary material for: The role of job strain in the relationship between depression and long-term sickness absence: a register-based cohort study
Source: Soc Psychiatry Psychiatr Epidemiol. 2024 Jun 25;59(11):2031–9. doi: 10.1007/s00127-024-02700-7 (PMC11522148; doi:10.1007/s00127-024-02700-7)
Supplement: Supplementary file 1 — Supplementary Material 1 [file 127_2024_2700_MOESM1_ESM.docx]

**Supplementary tables**

**Table S1.** Associations between depression treatment and psychiatric LTSA stratified by job type

|  |  | **Cases of LTSA** |  |  |
| --- | --- | --- | --- | --- |
| **Job strain** |  | **N (%)** | **Model 1** | **Model 2** |
| **Active job** | No depression | 50208 (5.93) | 1.00 | 1.00 |
|  | Antidepressants | 16442 (19.42) | 3.72 (3.65−3.79) | 3.07 (3.01−3.12) |
|  | In/outpatient care | 3554 (31.58) | 6.64 (6.42−6.87) | 5.35 (5.17−5.54) |
| **High strain** | No depression | 35910 (6.93) | 1.00 | 1.00 |
|  | Antidepressants | 12928 (20.33) | 3.37 (3.31−3.44) | 2.96 (2.90−3.02) |
|  | In/outpatient care | 3394 (32.67) | 5.93 (5.72−6.14) | 5.36 (5.17−5.55) |
| **Low strain** | No depression | 30743 (5.97) | 1.00 | 1.00 |
|  | Antidepressants | 12263 (18.79) | 3.59 (3.52−3.67) | 2.97 (2.91−3.04) |
|  | In/outpatient care | 3223 (31.65) | 6.62 (6.39−6.87) | 5.62 (5.42−5.83) |
| **Passive job** | No depression | 47055 (5.82) | 1.00 | 1.00 |
|  | Antidepressants | 20429 (18.51) | 3.59 (3.53−3.65) | 2.99 (2.94−3.04) |
|  | In/outpatient care | 6209 (30.78) | 6.51 (6.34−6.68) | 5.58 (5.43−5.73) |
| **Demands** |  |  |  |  |
| **High** | No depression | 86118 (6.31) | 1.00 | 1.00 |
|  | Antidepressants | 29370(19.81) | 3.59 (3.54−3.63) | 3.03 (2.99−3.07) |
|  | In/outpatient care | 6948 (32.11) | 6.37 (6.21−6.52) | 5.38 (5.25−5.52) |
| **Low** | No depression | 77798 (5.88) | 1.00 | 1.00 |
|  | Antidepressants | 32692 (18.61) | 3.59 (3.54−3.64) | 2.99 (2.95−3.03) |
|  | In/outpatient care | 9432 (31.07) | 6.53 (6.39−6.67) | 5.62 (5.50−5.74) |
| **Control** |  |  |  |  |
| **Low** | No depression | 82965 (6.25) | 1.00 | 1.00 |
|  | Antidepressants | 33357 (19.16) | 3.49 (3.44−3.53) | 2.99 (2.95−3.02) |
|  | In/outpatient care | 9603 (31.42) | 6.25 (6.12−6.38) | 5.52 (5.40−5.64) |
| **High** | No depression | 80951 (5.28) | 1.00 | 1.00 |
|  | Antidepressants | 28705 (19.14) | 3.66 (3.62−3.71) | 3.03 (2.99−3.07) |
|  | In/outpatient care | 6777 (31.62) | 6.65 (6.48−6.81) | 5.50 (5.36−5.64) |

LTSA: Long term sickness absence.

Model 1: Adjusted for age.

Model 2: Adjusted for age, gender, education, civil status, country of birth, unemployment prior to the start of follow-up, diagnosis of musculoskeletal disorders prior to the start of follow-up, physical workload prior to the start of follow-up.

LR test: Likelihood ratio test of interaction.

PAF: Population Attributable Fraction = Pc X (HR-1/HR) where Pc=proportion of LTSA cases exposed to depression treatment.

**Table S2.** Associations between depression treatment and LTSA stratified by job type among men

|  |  | **Cases of LTSA** |  |  |
| --- | --- | --- | --- | --- |
| **Job strain** |  | **N (%)** | **Model 1** | **Model 2** |
| **Active job** | No depression | 48738 (10.01) | 1.00 | 1.00 |
|  | Antidepressants | 8547 (25.02) | 2.77 (2.71−2.84) | 2.54 (2.49−2.60) |
|  | In/outpatient care | 1742 (36.60) | 4.77 (4.54−5.00) | 4.16 (3.96−4.36) |
| **High strain** | No depression | 42039 (16.98) | 1.00 | 1.00 |
|  | Antidepressants | 7276 (33.12) | 2.23 (2.17−2.28) | 2.06 (2.01−2.11) |
|  | In/outpatient care | 1775 (41.83) | 3.31 (3.16−3.48) | 2.94 (2.81−3.09) |
| **Low strain** | No depression | 39837 (16.19) | 1.00 | 1.00 |
|  | Antidepressants | 5921 (31.75) | 2.23 (2.17−2.29) | 2.01 (1.96−2.07) |
|  | In/outpatient care | 1420 (41.39) | 3.43 (3.26−3.62) | 2.98 (2.83−3.14) |
| **Passive job** | No depression | 86631 (18.64) | 1.00 | 1.00 |
|  | Antidepressants | 15235 (34.94) | 2.21 (2.17−2.24) | 2.00 (1.96−2.03) |
|  | In/outpatient care | 3911 (42.39) | 3.18 (3.08−3.29) | 2.85 (2.76−2.94) |
| **Demands** |  |  |  |  |
| **High** | No depression | 90777 (12.36) | 1.00 | 1.00 |
|  | Antidepressants | 15823 (28.19) | 2.57 (2.53−2.61) | 2.32 (2.28−2.36) |
|  | In/outpatient care | 3517 (39.06) | 4.21 (4.07−4.35) | 3.51 (3.39−3.63) |
| **Low** | No depression | 126468 (17.79) | 1.00 | 1.00 |
|  | Antidepressants | 21156 (33.99) | 2.23 (2.20−2.26) | 2.01 (1.98−2.04) |
|  | In/outpatient care | 5331 (42.12) | 3.28 (3.20−3.38) | 2.89 (2.81−2.97) |
| **Control** |  |  |  |  |
| **Low** | No depression | 128670 (18.06) | 1.00 | 1.00 |
|  | Antidepressants | 22511 (34.33) | 2.32 (2.30−2.34) | 2.09 (2.08−2.11) |
|  | In/outpatient care | 5686 (42.21) | 3.23 (3.18−3.29) | 2.94 (2.90−2.99) |
| **High** | No depression | 88575 (12.09) | 1.00 | 1.00 |
|  | Antidepressants | 14468 (27.40) | 2.64 (2.62−2.66) | 2.34 (2.32−2.36) |
|  | In/outpatient care | 3162 (38.60) | 4.09 (4.01−4.18) | 3.48 (3.41−3.55) |

LTSA: Long term sickness absence.

Model 1: Adjusted for age.

Model 2: Adjusted for age, education, civil status, country of birth, unemployment prior to the start of follow-up, diagnosis of musculoskeletal disorders prior to the start of follow-up, physical workload prior to the start of follow-up.

LR test: Likelihood ratio test of interaction.

PAF: Population Attributable Fraction = Pc X (HR-1/HR) where Pc=proportion of LTSA cases exposed to depression treatment.

**Table S3.** Associations between depression treatment and LTSA stratified by job type among women

|  |  | **Cases of LTSA** |  |  |
| --- | --- | --- | --- | --- |
| **Job strain** |  | **N (%)** | **Model 1** | **Model 2** |
| **Active job** | No depression | 69617 (19.33) | 1.00 | 1.00 |
|  | Antidepressants | 19847 (39.28) | 2.41 (2.38−2.45) | 2.23 (2.19−2.26) |
|  | In/outpatient care | 3343 (51.49) | 3.67 (3.55−3.80) | 3.29 (3.18−3.41) |
| **High strain** | No depression | 63562 (23.47) | 1.00 | 1.00 |
|  | Antidepressants | 18182 (43.67) | 2.24 (2.21−2.28) | 2.07 (2.04−2.11) |
|  | In/outpatient care | 3433 (55.87) | 3.36 (3.24−3.48) | 3.12 (3.01−3.23) |
| **Low strain** | No depression | 62221 (23.12) | 1.00 | 1.00 |
|  | Antidepressants | 20012 (42.94) | 2.22 (2.19−2.26) | 2.01 (1.98−2.04) |
|  | In/outpatient care | 3674 (54.42) | 3.36 (3.25−3.47) | 3.01 (2.91−3.11) |
| **Passive job** | No depression | 100404 (29.20) | 1.00 | 1.00 |
|  | Antidepressants | 33010 (49.38) | 2.07 (2.04−2.10) | 1.89 (1.87−1.91) |
|  | In/outpatient care | 6239 (56.99) | 2.80 (2.73−2.87) | 2.63 (2.57−2.70) |
| **Demands** |  |  |  |  |
| **High** | No depression | 133179 (21.11) | 1.00 | 1.00 |
|  | Antidepressants | 38029 (41.26) | 2.34 (2.31−2.37) | 2.16 (2.13−2.18) |
|  | In/outpatient care | 6776 (53.62) | 3.55 (3.46−3.63) | 3.22 (3.14−3.30) |
| **Low** | No depression | 162625 (26.53) | 1.00 | 1.00 |
|  | Antidepressants | 53022 (46.73) | 2.14 (2.12−2.16) | 1.93 (1.91−1.95) |
|  | In/outpatient care | 9913 (56.01) | 3.02 (2.96−3.08) | 2.76 (2.71−2.82) |
| **Control** |  |  |  |  |
| **Low** | No depression | 163966 (26.68) | 1.00 | 1.00 |
|  | Antidepressants | 51192 (47.19) | 2.32 (2.30−2.34) | 2.09 (2.08−2.11) |
|  | In/outpatient care | 9672 (56.58) | 3.23 (3.18−3.29) | 2.94 (2.90−2.99) |
| **High** | No depression | 131838 (20.95) | 1.00 | 1.00 |
|  | Antidepressants | 39859 (41.04) | 2.64 (2.61−2.66) | 2.34 (2.32−2.36) |
|  | In/outpatient care | 7017 (52.98) | 4.09 (4.01−4.18) | 3.48 (3.41−3.55) |

LTSA: Long term sickness absence.

Model 1: Adjusted for age.

Model 2: Adjusted for age, education, civil status, country of birth, unemployment prior to the start of follow-up, diagnosis of musculoskeletal disorders prior to the start of follow-up, physical workload prior to the start of follow-up.

LR test: Likelihood ratio test of interaction.

PAF: Population Attributable Fraction = Pc X (HR-1/HR) where Pc=proportion of LTSA cases exposed to depression treatment.

**Table S4.** Associations between depression treatment and LTSA stratified by job type, excluding those with previous LTSA

|  |  | **Cases of LTSA** |  |  |
| --- | --- | --- | --- | --- |
| **Job strain** |  | **N (%)** | **Model 1** | **Model 2** |
| **Active job** | No depression | 85022 (11.56) | 1.00 | 1.00 |
|  | Antidepressants | 10541 (23.59) | 2.21 (2.16-2.25) | 1.95 (1.91-1.99) |
|  | In/outpatient care | 1124 (30.08) | 3.01 (2.84-3.19) | 2.61 (2.46-2.77) |
| **High strain** | No depression | 70466 (16.77) | 1.00 | 1.00 |
|  | Antidepressants | 8770 (29.13) | 1.89 (1.85-1.93) | 1.76 (1.73-1.80) |
|  | In/outpatient care | 1040 (33.48) | 2.31 (2.18-2.46) | 2.16 (2.03-2.30) |
| **Low strain** | No depression | 67004 (16.12) | 1.00 | 1.00 |
|  | Antidepressants | 9026 (28.79) | 1.95 (1.91-1.99) | 1.74 (1.70-1.78) |
|  | In/outpatient care | 1075 (34.21) | 2.51 (2.36-2.67) | 2.22 (2.09-2.36) |
| **Passive job** | No depression | 119224 (18.85) | 1.00 | 1.00 |
|  | Antidepressants | 16245 (32.48) | 1.91 (1.88-1.95) | 1.72 (1.69-1.75) |
|  | In/outpatient care | 2044 (34.29) | 2.19 (2.10-2.29) | 2.03 (1.94-2.12) |
| **Demands** |  |  |  |  |
| **High** | No depression | 155488 (13.46) | 1.00 | 1.00 |
|  | Antidepressants | 19311 (25.82) | 2.08 (2.05-2.11) | 1.87 (1.84-1.90) |
|  | In/outpatient care | 2164 (31.62) | 2.72 (2.61-2.84) | 2.39 (2.29-2.49) |
| **Low** | No depression | 186228 (17.77) | 1.00 | 1.00 |
|  | Antidepressants | 25271 (31.05) | 1.93 (1.90-1.95) | 1.73 (1.70-1.75) |
|  | In/outpatient care | 3119 (34.26) | 2.31 (2.23-2.39) | 2.10 (2.02-2.17) |
| **Control** |  |  |  |  |
| **Low** | No depression | 189690 (18.02) | 1.00 | 1.00 |
|  | Antidepressants | 25015 (31.21) | 1.91 (1.88-1.93) | 1.73 (1.71-1.76) |
|  | In/outpatient care | 3084 (34.01) | 2.24 (2.16-2.32) | 2.07 (1.99-2.14) |
| **High** | No depression | 152026 (13.21) | 1.00 | 1.00 |
|  | Antidepressants | 19567 (25.73) | 2.12 (2.09-2.15) | 1.85 (1.82-1.87) |
|  | In/outpatient care | 2199 (31.97) | 2.83 (2.71-2.95) | 2.41 (2.31-2.51) |

LTSA: Long term sickness absence.

Model 1: Adjusted for age.

Model 2: Adjusted for age, gender, education, civil status, country of birth, unemployment prior to the start of follow-up, diagnosis of musculoskeletal disorders prior to the start of follow-up, physical workload prior to the start of follow-up.

PAF: Population Attributable Fraction = Pc X (HR-1/HR) where Pc=proportion of LTSA cases exposed to depression treatment.
